# Hypercapnia elicits differential vascular and blood flow responses in the cerebral circulation and active skeletal muscles in exercising humans

**DOI:** 10.14814/phy2.15274

**Published:** 2022-04-24

**Authors:** Shodai Moriyama, Masashi Ichinose, Kohei Dobashi, Ryoko Matsutake, Mizuki Sakamoto, Naoto Fujii, Takeshi Nishiyasu

**Affiliations:** ^1^ 13121 Faculty of Health and Sport Sciences University of Tsukuba Tsukuba City Ibaraki Japan; ^2^ 12939 Human Integrative Physiology Laboratory School of Business Administration Meiji University Tokyo Japan; ^3^ Faculty of Education Hokkaido University of Education Hokkaido Japan

**Keywords:** active skeletal muscle blood flow, cerebral blood flow, dynamic exercise, hypercapnia

## Abstract

The purpose of this study was to investigate the effects of a rise in arterial carbon dioxide pressure (PaCO_2_) on vascular and blood flow responses in the cerebral circulation and active skeletal muscles during dynamic exercise in humans. Thirteen healthy young adults (three women) participated in hypercapnia and normocapnia trials. In both trials, participants performed a two‐legged dynamic knee extension exercise at a constant workload that increased heart rate to roughly 100 beats min^−1^. In the hypercapnia trial, participants performed the exercise with spontaneous breathing while end‐tidal carbon dioxide pressure (P_ET_CO_2_), an index of PaCO_2_, was held at 60 mmHg by inhaling hypercapnic gas (O_2_: 20.3 ± 0.1%; CO_2_: 6.0 ± 0.5%). In the normocapnia trial, minute ventilation during exercise was matched to the value in the hypercapnia trial by performing voluntary hyperventilation with P_ET_CO_2_ clamped at baseline level (i.e., 40–45 mmHg) through inhalation of mildly hypercapnic gas (O_2_: 20.6 ± 0.1%; CO_2_: 2.7 ± 1.0%). Middle cerebral artery mean blood velocity and the cerebral vascular conductance index were higher in the hypercapnia trial than in the normocapnia trial. By contrast, vascular conductance in the exercising leg was lower in the hypercapnia trial than in the normocapnia trial. Blood flow to the exercising leg did not differ between the two trials. These results demonstrate that hypercapnia‐induced vasomotion in active skeletal muscles is opposite to that in the cerebral circulation. These differential vascular responses may cause a preferential rise in cerebral blood flow.

## 1 INTRODUCTION

The cerebrovascular tone is significantly affected by changes in the arterial carbon dioxide pressure (PaCO_2_) (Battisti‐Charbonney et al., 2011; Coverdale et al., 2014; Ide et al., 2003; Ogoh et al., 2008, 2009; Rasmussen et al., 2006; Subudhi et al., 2011). In response to an increase or decrease in PaCO_2_ (hypercapnia or hypocapnia, respectively), cerebral vasodilation or vasoconstriction occurs to maintain pH in the cerebrospinal fluid (Ogoh et al., 2008, 2009). For instance, in resting humans, cerebral blood flow increases by approximately 4% per mmHg end‐tidal carbon dioxide pressure (P_ET_CO_2_, an index of PaCO_2_) in the hypercapnic range and decreases by approximately 2% per mmHg P_ET_CO_2_ in the hypocapnic range (Ide et al., 2003).

Changes in PaCO_2_ also affect peripheral blood vessels, although the effects are less clear. Several studies performed in resting humans found that hypercapnia had no effect on blood flow or vascular resistance in the peripheral circulation (Kontos et al., 1968a; Xie et al., 2001), whereas other studies observed that hypercapnia elicited a decrease in blood flow to the hand (Gellhorn & Steck, 1938) or an increase in brachial blood flow (Vantanajal et al., 2007). Thus, there is no consensus regarding the effect of hypercapnia on peripheral circulatory responses.

During dynamic exercise, the pattern of blood flow to the active and inactive skeletal muscles, brain, and other organs is determined by local metabolic and myogenic factors and neuronally mediated vasoconstriction (Laughlin et al., 1996). Blood flow in active skeletal muscles accounts for nearly all of the increased cardiac output (CO) during dynamic exercise (Rowell, 1993). Consequently, the impact of vasomotion within active skeletal muscles on systemic arterial pressure and hemodynamics during dynamic exercise is much greater than that in inactive skeletal muscles at rest. Hypercapnia is a key element driving local vasodilation in both the peripheral (Kontos et al., 1968b) and cerebral vessels (Al‐Khazraji et al., 2019; Coverdale et al., 2014). Moreover, hypercapnia elevates sympathetic nerve activity (Ainslie et al., 2005; Jouett et al., 2015; Narkiewicz et al., 1999; Somers et al., 1989, 1991; Toledo et al., 2017) by activating central and peripheral chemoreflexes (Guyenet et al., 2010; Moreira et al., 2006; Schultz & Sun, 2000; Toledo et al., 2017). Thus, hypercapnia during dynamic exercise may alter blood flow distribution and systemic arterial pressure by changing cerebrovascular and active skeletal muscle vascular tone. This means that cerebral and peripheral circulatory responses to hypercapnia during dynamic exercise should be measured simultaneously in the same individual. However, no such studies have yet been conducted, although they have been investigated in humans at rest (Lennox & Gibbs, 1932; Vantanajal et al., 2007) and during static handgrip exercise (Ainslie et al., 2005).

To the best of our knowledge, a recent study by Wan et al. (2020) is the first to examine peripheral circulatory responses to hypercapnia (PaCO_2_: ~50 mmHg) during dynamic exercise. They reported that exercising leg blood flow (LBF) and leg vascular conductance (LVC) were unaffected by hypercapnia. However, considering that muscle sympathetic nerve activity increases in proportion to the increase in P_ET_CO_2_ (Jouett et al., 2015), sympathetic vasoconstriction in active skeletal muscles may occur when employing a greater magnitude of hypercapnia relative to the level employed in the study by Wan et al. (2020). In addition, given that hypercapnia‐induced increase in arterial pressure is involved in elevated middle cerebral artery mean blood velocity (MCAV_mean_: an index of cerebral blood flow) when cerebral autoregulation is exhausted (Battisti‐Charbonney et al., 2011), hypercapnia‐induced pressor response associated with sympathetic vasoconstriction in active skeletal muscles may be involved in the cerebral blood flow response during dynamic exercise.

Therefore, the purpose of the present study was to investigate the effects of hypercapnia in the range of PaCO_2_ > 50 mmHg on vascular and blood flow responses in the cerebral circulation and active skeletal muscles during dynamic exercise in humans. To achieve this purpose, we conducted simultaneous and continuous measurements of MCAV_mean_ and femoral artery blood flow (LBF) during two‐legged dynamic knee extension exercise with and without hypercapnia. We hypothesized that hypercapnia causes cerebral vasodilation but induces vasoconstriction within active skeletal muscle.

## 2 MATERIALS AND METHODS

### 2.1 Participants

Ten healthy men and three healthy women participated in this study. The participants were aged 23 ± 3 (mean ± standard deviation) years, with 1.71 ± 0.08 m in height, and weighed 68.8 ± 8.8 kg. None of the participants were smokers nor taking prescription medications. All participants refrained from caffeine and alcohol for >24 h and food for 2 h prior to the experiments, and were instructed to avoid intense exercise the night before evaluation. Female participants participated in the experiments during their early follicular phase to minimize the influence of increases in estrogen or progesterone levels on circulatory responses (Wallace et al., 2010).

### 2.2 Preliminary session

All participants engaged in a preliminary session to become familiar with the two‐legged dynamic knee extension exercise. In addition, the workload of knee extension exercise to be used in the experimental session and the target tidal volume (V_T_) to be used during voluntary hyperventilation in the experimental session (see below) were determined.

### 2.3 Experimental protocol

On the day of the experiment, the participants entered the test room (room temperature: 24.3 ± 1.2°C) and adopted a semi‐supine position on the ergometer. After the equipment was set up, the participants maintained their resting position for 3 min, during which the femoral artery blood flow was measured in the right leg (rest). Thereafter, the participants started a 10‐min knee extension exercise (60 rpm) at a constant workload (Ex baseline) that increased the heart rate (HR) to roughly 100 beats min^−1^, as determined in the preliminary session. This exercise modality and intensity allowed us to measure the femoral artery blood flow during exercise. The average workload was 38 ± 10 W. Five minutes after starting the exercise, the participants began a 5‐min period of CO_2_ inhalation (CO_2_ inhalation: details will be described in the “CO_2_ inhalation” section). This protocol was performed under two conditions: (1) normoxic normocapnia (normocapnia trial) and (2) normoxic hypercapnia (hypercapnia trial). During the experiment, a fan was directed at the participant’s legs to minimize leg cutaneous vasodilation mediated by exercise‐induced increases in body temperature (Simmons et al., 2011). Both trials were performed on the same day and separated by at least 20‐min. We randomized and counterbalanced the order of the trials.

### 2.4 CO_2_ inhalation

During the CO_2_ inhalation period in the hypercapnia trial, the participants exercised with spontaneous breathing [i.e., their V_T_ and respiratory frequency (*f*_R_) were not controlled], while P_ET_CO_2_ was held at 60 mmHg by inhalation of hypercapnic gas (O_2_: 20.3 ± 0.1%; CO_2_: 6.0 ± 0.5%). The experimenter monitored the breath‐by‐breath P_ET_CO_2_ data obtained from the mass spectrometer and manually adjusted the CO_2_ flow rate using a gas flow meter (RK1150, KOFLOC, Japan) to maintain P_ET_CO_2_ levels. To minimize the potential difference in the work of breathing between the two trials, minute ventilation (V_E_) during CO_2_ inhalation in the normocapnia trial was matched to the value during CO_2_ inhalation in the hypercapnia trial, wherein the *f*_R_ was set at 60 breaths min^−1^ so that the participants could easily match the timing of their kicking and breathing. The V_T_ level employed in the normocapnia trial was predetermined in the preliminary session, as noted above. The respiratory pattern used in the normocapnia trial was accomplished using visual feedback from a computer display showing V_T_ and auditory cues from a metronome for *f*_R_. To prevent the reduction in P_ET_CO_2_ caused by voluntary hyperventilation (Chin et al., 2013; Dobashi et al., 2017), the participants inhaled mildly hypercapnic gas (O_2_: 20.6 ± 0.1%; CO_2_: 2.7 ± 1.0%) simultaneously. In this way, P_ET_CO_2_ was maintained at the Ex baseline level (i.e., 40–45 mmHg) in the normocapnia trial. Similar to the hypercapnia trial, the CO_2_ flow rate was manually adjusted by the experimenter in the normocapnia trial.

### 2.5 Measurements

The participants breathed from a low‐dead space mask that covered their nose and mouth. A pneumotachograph transducer for evaluating respiratory volume was attached to the mask and a gas‐sampling tube (sampling rate of 60 ml min^−1^) was attached to the pneumotachograph transducer. Respiratory variables were assessed using a mass spectrometer (ARCO‐1000, Arco System, Japan), which analyzed respiratory O_2_ and CO_2_ pressures. Before starting the measurements, the mass spectrometer was calibrated using reference gases of known concentrations (O_2_: 15.1%, CO_2_: 5.0%). The flow sensor was calibrated using an appurtenant calibration syringe able to blow a fixed volume (3 L) of air. The mass spectrometer provided breath‐to‐breath V_E_ and P_ET_CO_2_ values based on the measured respiratory volume and/or gases.

The HR was monitored using a three‐lead electrocardiogram (ECG). Beat‐to‐beat changes in arterial blood pressure were assessed using finger photoplethysmography (Finometer, Finapres Medical Systems). The cuff was placed around the middle finger of the left hand, with the forearm and hand supported so that the cuff was aligned at the level of the heart. We estimated the beat‐to‐beat stroke volume (SV) from the blood pressure waveform using the Modelflow software (Wesseling et al., 1993). CO was calculated as the product of SV and HR. Total vascular conductance (TVC) was then calculated as CO/mean arterial pressure (MAP).

We measured LBF using Doppler ultrasound, as previously described (Ichinose et al., 2018; Ichinose & Nishiyasu, 2005; Nishiyasu et al., 2012). A Doppler ultrasound system (iU‐22; Philips, USA) equipped with a handheld transducer probe (model L12‐5) with an operating frequency of 6 MHz was utilized to simultaneously measure the two‐dimensional common femoral artery diameter and mean blood velocity (MBV). Measurements were performed by positioning the transducer probe over the common femoral artery in the right thigh, 2–3 cm distal to the inguinal ligament. All Doppler data were recorded continuously on an S‐VHS videotape (ST‐120; Maxell, Japan). The videotape record of the vessel image was digitized using a digital video board (PCI‐1411; National Instruments, USA) and stored on a personal computer equipped with software to measure the vessel diameter. During the last 1 min of the rest, Ex baseline, and CO_2_ inhalation periods, the largest and smallest femoral artery diameters within each cardiac cycle were measured for five heartbeats, and the mean values of each were defined as the systolic (Ds; mm) and diastolic (Dd; mm) diameters, respectively. The mean diameter (Dm; mm) was calculated as Dm = Ds/3 + 2 × Dd/3. The femoral artery cross‐sectional area (CSA) was estimated using a representative Dm, using the formula: CSA = (Dm/10/2)^2^ π.

Instantaneous MBV was continuously estimated using a computer program developed with the aid of LabVIEW (version 6.0; National Instruments, USA). The processes used in the MBV calculations are outlined below: The frequency spectrum of the analog audio output signal of our ultrasound Doppler unit robustly reflects the Doppler shift frequency spectrum within the audio range (7.5 kHz in this study). The analog audio output signal was digitized at a sampling frequency of 20 kHz using an analog‐to‐digital converter (DAQCard‐6062E, National Instruments) for processing on a personal computer (ThinkPad T30, IBM) equipped with our program. The power spectrum of the digitized audio signal was obtained using fast Fourier transform analysis techniques using a Hanning smoothing window with a 512‐data point segment. The mean frequency of a data segment (*f*_me_) was derived from the spectral data using the following equation:

$$f_{\text{me}}=\frac{\sum_{i=0}^{N/2}\left(f_{i}\cdot P_{i}\right)}{\sum_{i=0}^{N/2}f_{i}}.$$

where *f_i_* is the spectral frequency, *P_i_* is the power related to *f_i_*, and *N* is the number of data points. We began an analysis of the next data segment of the digitized audio signal, which was advanced to 200 data points from the beginning of the previous segment so that the 312 data points (15.6 ms) overlapped. Our program repeated the above processes in real‐time and continuously produced 100 values of *f*_me_ per second (100 Hz). The *f*_me_ calculated using the above processes correlated well with the actual mean Doppler shift frequency when the electrically generated arbitrary ultrasound wave was transmitted to the transducer probe and measured using our ultrasound Doppler unit. Therefore, we regarded *f*_me_ as the mean Doppler shift frequency and used it to calculate instantaneous MBV. The analog signals representing the ECG and blood pressure waveform were digitized at a sampling frequency of 100 Hz and stored together with *f*_me_ (thus, all data could be analyzed together for the same time period). The HR, systolic blood pressure, diastolic blood pressure, MAP, and MBV were calculated using an offline data analysis program. The MBV was derived from the stored *f*_me_ data using the following equation:

$$\text{MBV}=\frac{f_{\text{me}}\times C}{2\times f_{\text{e}}\times \cos60^{\circ }}\times 100.$$

where *f*_e_ is the emitted frequency from the transducer probe (6 MHz for femoral and brachial blood flow and 2 MHz for aortic blood flow) and *C* is the sound velocity in the tissues (1,530 m sec^−1^). We applied the above formula to all the stored *f*_me_ data and obtained an instantaneous MBV profile over the entire measurement period. The instantaneous MBV profile was then integrated over each cardiac cycle to acquire the beat‐by‐beat velocity‐time integral (VTI; in cm beat^−1^). LBF was then calculated as CSA × VTI × HR. The LVC was calculated as LBF/MAP.

MCAV_mean_ was determined using a transcranial Doppler ultrasound device (EZ‐Dop; Compumedics, Singen, Germany), as previously described (Fujii et al., 2019, 2021). Briefly, a 2‐ MHz Doppler probe was affixed to the temporal bone. The middle cerebral artery was insonated at a depth of 42–59 mm from the temporal bone. MCAV_mean_ was continuously recorded at a sampling rate of 200 Hz and stored on a computer via a data acquisition system (Power Lab; ADInstruments, Australia). The cerebral vascular conductance index (CVCi) was calculated as MCAV_mean_/MAP × 100.

Ratings of perceived effort of breathing (range, 0–10) (Borg, 1982) were recorded immediately after exercise.

### 2.6 Data analysis

Circulatory and respiratory variables were averaged over the last 1 min of the rest, Ex baseline, and CO_2_ inhalation periods. In response to hypercapnia, both local cerebral vasodilation and elevation in cerebral perfusion pressure could increase cerebral blood flow (Battisti‐Charbonney et al., 2011). Therefore, we estimated the relative contributions of cerebral vasodilation and increased arterial pressure to hypercapnia‐induced increases in MCAV_mean_ during dynamic exercise, using the following equations:

$$\begin{array}{c} {\text{MCAV}}_{\text{mean}}\text{estimated}=\text{MAP}\;\text{in}\;\text{normocapnia}\\ \;\times \text{CVCi}\;\text{in}\;\text{hypercapnia} \end{array}$$

$$\begin{array}{c} \Delta {\text{MCAV}}_{\text{mean}}\text{estimated}={\text{MCAV}}_{\text{mean}}\;\text{estimated}\\ \;‐{\text{MCAV}}_{\text{mean}}\text{in}\;\text{normocapnia} \end{array}$$

$$\begin{array}{c} \begin{array}{c} \%\;\text{Contribution}\;\text{of}\;\text{vasodilation}\\ =\left[\Delta {\text{MCAV}}_{\text{mean}}\text{estimated}/({\text{MCAV}}_{\text{mean}}\text{in}\;\text{hypercapnia}\right.\\ \left.‐{\text{MCAV}}_{\text{mean}}\text{in}\;\text{normocapnia})\times 100\right] \end{array} \end{array}$$

$$\begin{array}{c} \%\;\text{Contribution}\;\text{of}\;\text{increased}\;\text{arterial}\;\text{pressure}\\ \;=100‐\%\;\text{contribution}\;\text{of}\;\text{vasodilation} \end{array}$$

where MAP in normocapnia, CVCi in hypercapnia, and MCAV_mean_ in normocapnia are all data during CO_2_ inhalation; MCAV_mean_ estimated is the estimated level of MCAV_mean_ under hypercapnia if only cerebral vasodilation occurs; ∆MCAV_mean_ estimated is the estimated amount of increase in MCAV_mean_ caused by cerebral vasodilation; % contribution of vasodilation is the relative contribution (%) of cerebral vasodilation to the hypercapnia‐induced increase in MCAV_mean_; and % contribution of increased arterial pressure is the relative contribution (%) of increased arterial pressure to increased MCAV_mean_.

### 2.7 Statistical analysis

The minimal sample size was calculated on the basis of previously collected data evaluating hypercapnia‐induced changes in forearm blood flow (CO_2_ breathing vs. Control: 3.5 ± 0.3 vs. 3.0 ± 0.3 ml min^−1^ per 100 ml) (Kontos et al., 1968a) and in MCAV_mean_ (during CO_2_ breathing vs. pre‐CO_2_ breathing: 75.6 ± 16.1 vs. 62.6 ± 11.7 cm sec^−1^) (Panerai et al., 1999) in resting humans. We determined that a minimum of 6 and 12 participants would be required for forearm blood flow and MCAV_mean_, respectively, with 80% power and an α level of 0.05. The normal distribution of variables was checked using the Shapiro–Wilk test. Two‐way ANOVA was used to analyze the variables; the factors were trial (normocapnia and hypercapnia) and time (rest, Ex baseline, and CO_2_ inhalation). When a main effect of time or interaction was detected, post hoc multiple comparisons were performed with Bonferroni correction. For variables that were not normally distributed (i.e., *f*_R_, LBF, and LVC), the Friedman test (nonparametric test) was used to examine the effect of time (rest, Ex baseline, and CO_2_ inhalation) in each trial (normocapnia and hypercapnia). When the effect of time was detected, the Wilcoxon test (nonparametric test) was used for pairwise comparison. The Wilcoxon test was also used to compare normocapnia and hypercapnia at each time point (rest, Ex baseline, and CO_2_ inhalation). The student's paired *t*‐test was used to compare the relative contributions of cerebral vasodilation and increased arterial pressure to the hypercapnia‐induced increase in the MCAV_mean_. The Shapiro–Wilk test revealed that the perceived effort of breathing was not normally distributed. Therefore, the Wilcoxon test was used for pairwise comparison of the perceived effort of breathing. Statistical significance was set at *p* < 0.05. The data for which nonparametric tests were used are expressed as median values, and other data are expressed as mean ± standard deviation. The statistical software package SPSS 27 for Windows (IBM, Armonk, NY, USA) was used for all the statistical analyses.

## 3 RESULTS

**FIGURE 1:**
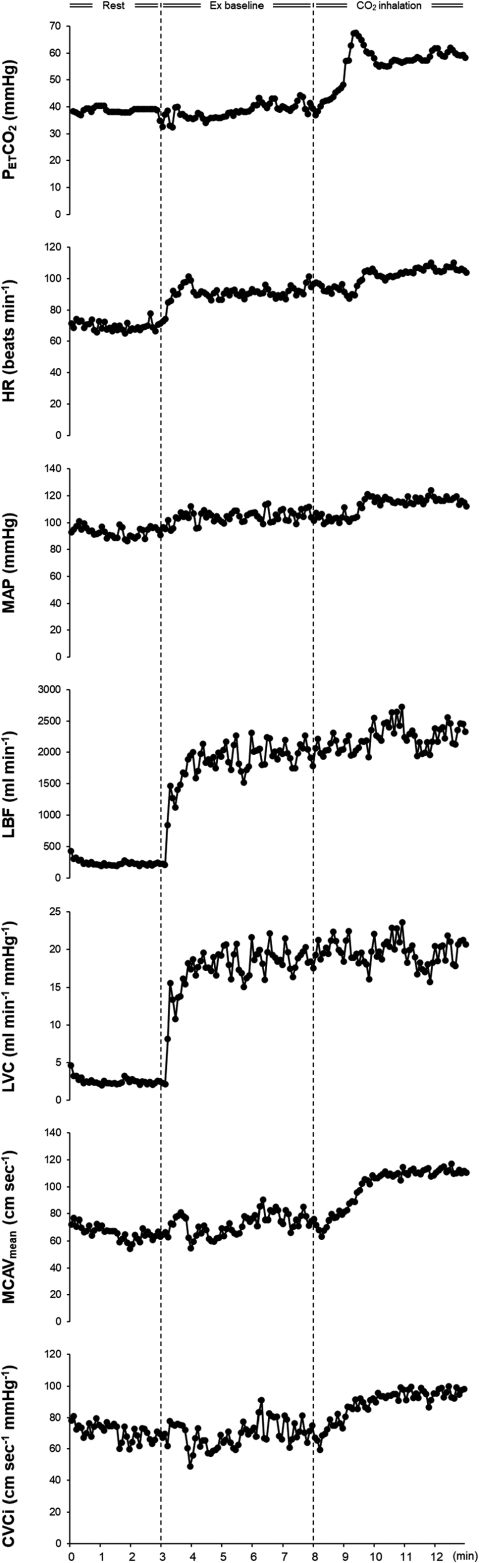
End‐tidal carbon dioxide pressure (P_ET_CO_2_), heart rate (HR), mean arterial pressure (MAP), leg blood flow (LBF), leg vascular conductance (LVC), middle cerebral artery mean blood velocity (MCAV_mean_), and cerebral vascular conductance index (CVCi) responses recorded from a representative participant in the hypercapnia trial. All data were averaged over 5‐sec intervals

**FIGURE 2:**
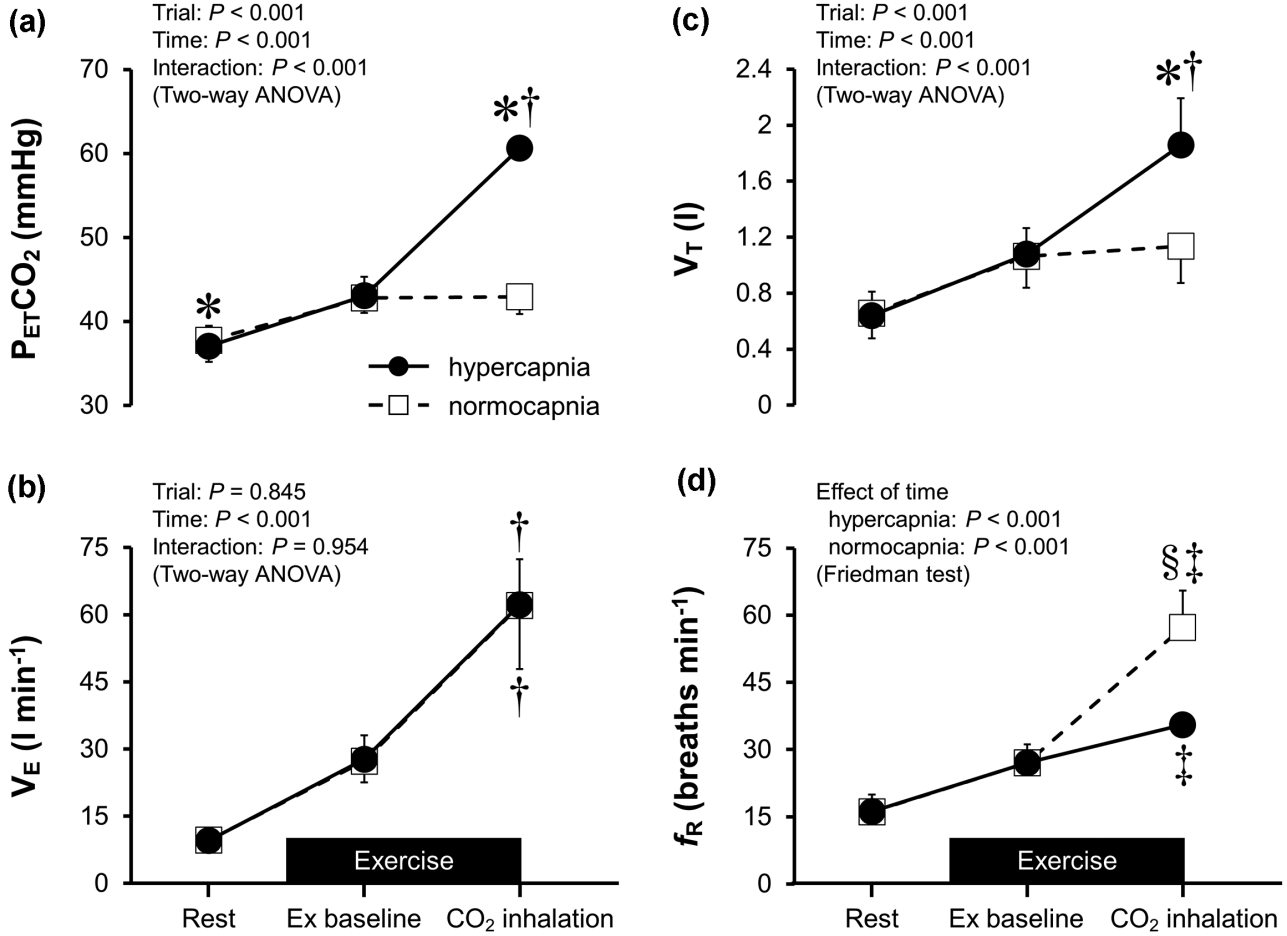
Time‐dependent changes in end‐tidal carbon dioxide pressure (P_ET_CO_2_, panel a), minute ventilation (V_E_, panel b), tidal volume (V_T_, panel c), and respiratory frequency (*f*_R_, panel d) during the rest and exercise periods. Ex baseline, constant workload exercise with spontaneous breathing; CO_2_ inhalation, CO_2_ was inhaled to induce hypercapnia during the exercise in the hypercapnia trial. CO_2_ was also inhaled in the normocapnia trial, but hypercapnia was prevented by voluntary hyperventilation. *p < 0.05 vs. the normocapnia trial assessed with Bonferroni's test; ^†^ *p* < 0.05 vs. Ex baseline assessed with Bonferroni's test; ^§^ *p* < 0.05 vs. the normocapnia trial assessed with the Wilcoxon test; ^‡^ *p* < 0.05 vs. Ex baseline assessed with the Wilcoxon test. Data are means ± standard deviation

Figure 1 shows the P_ET_CO_2_ and circulatory responses recorded from a representative participant during the hypercapnia trial. Time‐dependent changes in respiratory variables during the rest and exercise periods are illustrated in Figure 2, while the data for *f*_R_ for which nonparametric tests were used are also shown in Table 1. At rest, V_T_ and *f*_R_ were similar between the two trials, whereas P_ET_CO_2_ was lower in the hypercapnia trial than in the normocapnia trial (37.0 ± 2.4 mmHg vs. 37.7 ± 2.6 mmHg, *p* = 0.033), although the difference was not physiologically important. At Ex baseline, there were no between‐trial differences in P_ET_CO_2_, V_T_ and *f*_R_. By design, P_ET_CO_2_ during CO_2_ inhalation was higher in the hypercapnia trial than in the normocapnia trial (60.6 ± 1.3 vs. 42.9 ± 2.0 mmHg, *p* < 0.001). In the normocapnia trial, P_ET_CO_2_ during CO_2_ inhalation was similar to that measured during Ex baseline (42.9 ± 2.0 vs. 42.8 ± 1.7 mmHg, *p* = 1.000).

**TABLE 1:** Results of the Friedman and Wilcoxon tests of *f*_R_, LBF and LVC

Variables	Rest	Exercise		*p*values for the Friedman test
		Ex baseline	CO_2_ inhalation	Effect of time
*f*_R_, breaths min^−1^				
Hypercapnia	17 [7,21]	27 [16, 36]	34 [22, 57]* ^,^ **	<0.001
Normocapnia	16 [7, 22]	28 [20, 32]	60 [30, 60]**	<0.001
LBF, ml min^−1^				
Hypercapnia	449 [211, 810]	2431 [1718, 3876]	2490 [1567, 3764]	<0.001
Normocapnia	510 [210, 698]	2650 [1610, 3715]	2721 [2029, 2957]	<0.001
LVC, ml min^−1^ mmHg^−1^				
Hypercapnia	5.0 [2.4, 10.1]	23.1 [16.2, 41.3]	21.9 [12.7, 37.7]*	<0.001
Normocapnia	5.4 [2.3, 8.9]	24.4 [13.1, 37.0]	25.6 [17.9, 32.4]	<0.001

Note

Values are presented in median [minimum, maximum].

Abbreviations: *f*_R_, respiratory frequency; LBF, leg blood flow; LVC, leg vascular conductance.

* *p* < 0.05 vs. normocapnia trial assessed by the Wilcoxon test

** *p* < 0.05 vs. Ex baseline assessed by the Wilcoxon test.

**FIGURE 3:**
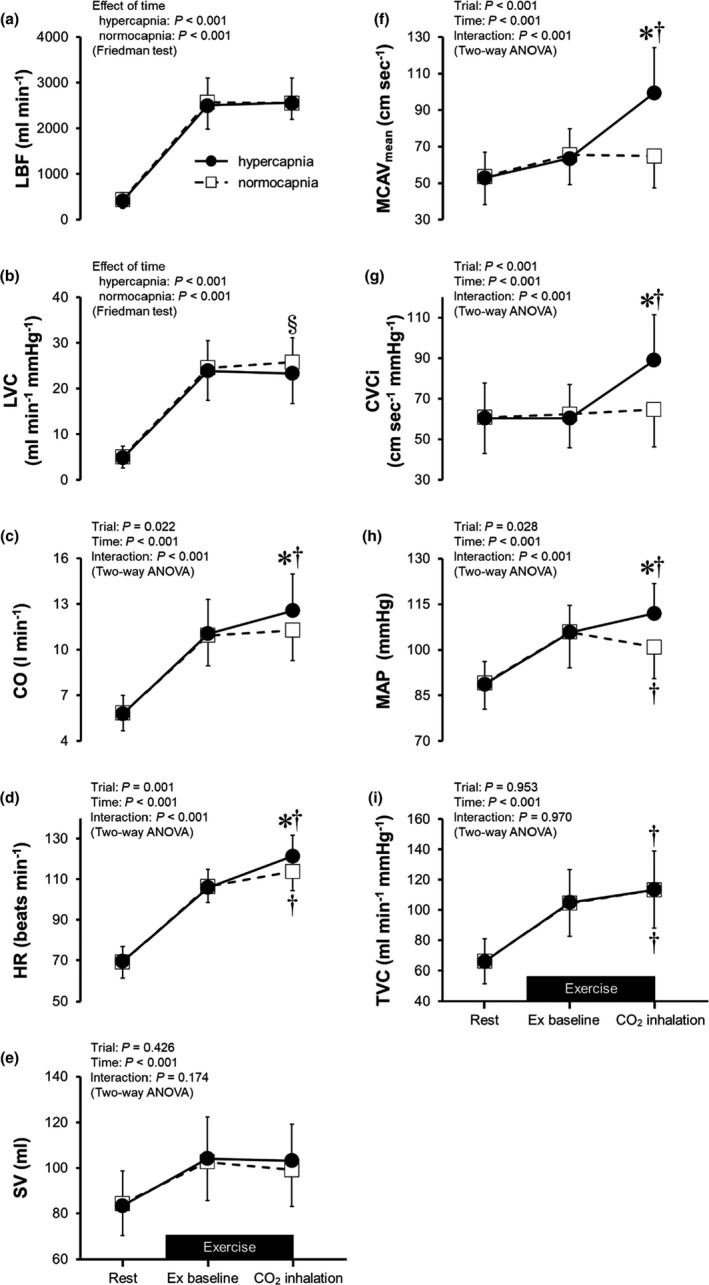
Time‐dependent changes in leg blood flow (LBF, panel a), leg vascular conductance (LVC, panel b), cardiac output (CO, panel c), heart rate (HR, panel d), stroke volume (SV, panel e), middle cerebral artery mean blood velocity (MCAV_mean_, panel f), cerebral vascular conductance index (CVCi, panel g), mean arterial pressure (MAP, panel h), and total vascular conductance (TVC, panel i) during the rest and exercise periods. Ex baseline, constant workload exercise with spontaneous breathing; CO_2_ inhalation, CO_2_ was inhaled to induce hypercapnia during the exercise in the hypercapnia trial. CO_2_ was also inhaled in the normocapnia trial, but hypercapnia was prevented by voluntary hyperventilation. *p < 0.05 vs. the normocapnia trial assessed with Bonferroni's test; ^†^ *p* < 0.05 vs. Ex baseline assessed with Bonferroni's test; ^§^ *p* < 0.05 vs. the normocapnia trial assessed with the Wilcoxon test. Data are means ± standard deviation

The time‐dependent changes in circulatory variables during the rest and exercise periods are presented in Figure 3, while the data for LBF and LVC for which nonparametric tests were used are also shown in Table 1. The data for MBV and Dm in femoral artery are shown in Table 2. At rest and at Ex baseline, none of the circulatory variables differed between the two conditions. During CO_2_ inhalation, both MCAV_mean_ (99 ± 25 vs. 65 ± 18 cm sec^−1^, *p* < 0.001) and CVCi (89 ± 22 vs. 65 ± 18 cm sec^−1^ mmHg^−1^, *p* < 0.001) were higher in the hypercapnia trial than in the normocapnia trial. By contrast, LVC was lower in the hypercapnia trial than in the normocapnia trial (median: 21.9 vs. 25.6 ml min^−1^ mmHg^−1^, *p* = 0.028). LBF did not differ between the hypercapnia and normocapnia trials (median: 2490 vs. 2721 ml min^−1^, *p* = 0.807). CO, HR, and MAP were higher in the hypercapnia trial than in the normocapnia trial during CO_2_ inhalation. TVC during CO_2_ inhalation was higher than the Ex baseline values in both trials, whereas SV during CO_2_ inhalation did not significantly differ from the Ex baseline value in either trial.

**TABLE 2:** Results of MBV and Dm in femoral artery

Variables	Rest	Exercise		*p* values for two‐way ANOVA		
		Ex baseline	CO_2_ inhalation	Main effect of trial	Main effect of time	Interaction
MBV, cm sec^−1^						
Hypercapnia	8.4 ± 3.2	48.2 ± 9.9	48.3 ± 10.8	0.847	<0.001	0.856
Normocapnia	8.9 ± 3.8	49.1 ± 11.6	47.9 ± 7.9			
Dm, mm						
Hypercapnia	10.2 ± 0.8	10.5 ± 0.8	10.6 ± 0.7	0.514	<0.001	0.834
Normocapnia	10.2 ± 0.8	10.6 ± 0.7	10.7 ± 0.7			

Note

Values are presented in means ± standard deviation.

Abbreviations: Dm, mean diameter; MBV, mean blood velocity.

**FIGURE 4:**
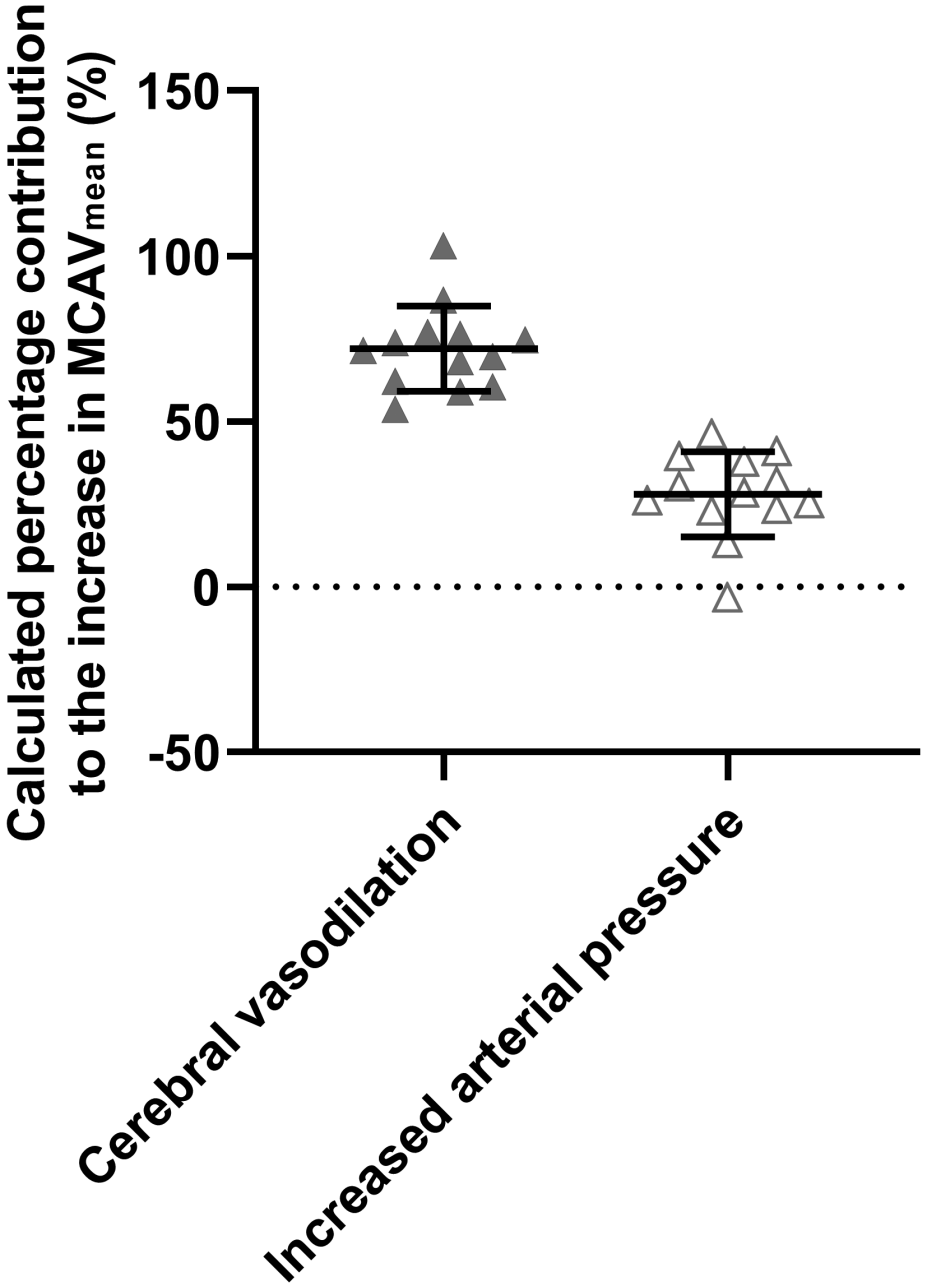
Calculated percentage contributions to the hypercapnia‐induced increase in MCAV_mean_ during dynamic exercise. See the “Data analysis” section for details on how the calculations were made. Shown are individual values and group means ± standard deviation

The calculated percentage contribution of cerebral vasodilation to the increase in MCAV_mean_ elicited by hypercapnia was significantly higher than that made by the increase in arterial pressure (72 ± 13% vs. 28 ± 13%, *p* < 0.001) (Figure 4).

The perceived effort of breathing recorded immediately after exercise was higher in the hypercapnia trial than in the normocapnia trial (5 ± 1 vs. 4 ± 2 a.u., *p* = 0.030).

## 4 DISCUSSION

We investigated the effects of hypercapnia on circulatory responses during a dynamic two‐legged knee extension exercise. We demonstrated that during CO_2_ inhalation, which results in increased arterial CO_2_ pressure, MCAV_mean_ and CVCi were higher, while LVC was lower in the hypercapnia trial than in the normocapnia trial. This suggests that hypercapnia during dynamic exercise elicits robust vasodilation in cerebral circulation while inducing vasoconstriction within active skeletal muscles.

We showed that hypercapnia markedly increased MCAV_mean_ and CVCi during dynamic exercise, which is consistent with previously reported results (Ogoh et al., 2008, 2009; Subudhi et al., 2011). By contrast, we found that hypercapnia decreased LVC during exercise, which contradicts a recent study by Wan et al. (2020), who reported that hypercapnia had no effect on LVC during a one‐legged dynamic knee extension exercise. This discrepancy may be attributable to the difference in the degree of hypercapnia between the present study (P_ET_CO_2_: 60.6 ± 1.3 mmHg) and that by Wan et al. (2020) (PaCO_2_: 50 ± 2 mmHg). Since hypercapnia elevates sympathetic nerve activity (Ainslie et al., 2005; Narkiewicz et al., 1999; Somers et al., 1989, 1991; Toledo et al., 2017) in a dose‐dependent manner (Jouett et al., 2015), the impact of sympathetic vasoconstriction may be greater in the present study than in that by Wan et al. (2020), overriding the local vasodilator effects of CO_2_ within active skeletal muscles. Despite the lower LVC in the hypercapnia trial than that in the normocapnia trial, there was no difference in LBF between the two trials (Table 1, Figure 3), which is consistent with the results reported by Wan et al. (2020). Given that MAP was higher in the hypercapnia trial than in the normocapnia trial (Figure 3), no change in LBF may reflect that the hypercapnia‐induced increase in perfusion pressure offset reduction in blood flow associated with sympathetic vasoconstriction. Notewhorthy, our study is the first to simultaneously measure brain and active skeletal muscle vascular responses to hypercapnia during dynamic exercise suggesting that cerebral and active skeletal muscle vessels exhibit different responses to hypercapnia.

The differential pattern of vascular responses between the two vascular beds may be explained by mechanisms associated with CO_2_ reactivity. Hypercapnia mediates robust cerebral vasodilation but elicits only minor vasodilation in the femoral (Ainslie et al., 2005) and brachial (Vantanajal et al., 2007) vessels. In line with this, Ainslie et al. (2005) reported that the CO_2_ reactivity in the femoral circulation is about eightfold lower than that in the cerebral circulation, although the mechanisms underlying region‐specific differences have not been fully elucidated. The differential vascular response to hypercapnia between the brain and active skeletal muscle also partly appears to be due to differential sympathetic innervation between the two vascular beds. It has been reported that fewer α‐adrenergic receptors are distributed in cerebral than peripheral blood vessels (Faraci & Heistad, 1998). Indeed, Ainslie et al. (2005) reported that handgrip exercise‐induced increases in sympathetic nerve activity did not change the MCAV_mean_. In addition, LeMarbre et al. (2003) reported that cerebrovascular CO_2_ reactivity in resting humans is unaffected by increased sympathetic nerve activity achieved through the application of lower body negative pressure. Therefore, sympathetic vasoconstriction associated with hypercapnia appears to be weaker in cerebral blood vessels, enabling marked cerebral vasodilation. By contrast, in active skeletal muscles, robust sympathetic vasoconstriction appears to mask any local vasodilator effect associated with hypercapnia during dynamic exercise.

Battisti‐Charbonney et al. (2011) reported that an increase in cerebral blood flow seen in response to hypercapnia (PaCO_2_ >50 mmHg) is achieved through both local cerebral vasodilation and elevation in cerebral perfusion pressure, the latter of which is attributable to elevation in arterial pressure via chemoreflexes. The effect of cerebral perfusion pressure on cerebral blood flow is augmented during hypercapnia, as dynamic cerebral autoregulation is attenuated during hypercapnia at rest (Aaslid et al., 1989; Maggio et al., 2013; Panerai et al., 1999). Although these findings were obtained in resting humans, hypercapnia‐induced attenuation of cerebral autoregulation may similarly occur during dynamic exercise, given that low‐intensity dynamic exercise does not affect dynamic cerebral autoregulation (Brys et al., 2003). Therefore, it is plausible that the elevations in MAP, induced by hypercapnia, observed in the present study contributed to the profound increase in MCAV_mean_. Based on our calculations, approximately 28 ± 13% of the hypercapnia‐induced increase in MCAV_mean_ was explained by increased arterial pressure. The observed increase in CO in the hypercapnia versus normocapnia trial could partially explain the increase in MAP in the hypercapnia trial. Moreover, it is noteworthy that TVC remained unchanged despite the local vasodilatory effects of CO_2_. This may be due, in part, to the vasoconstriction in exercising legs counteracting the local vasodilator effect of CO_2_, ultimately resulting in no between‐trial differences in TVC. The unchanged TVC, in conjunction with elevated CO, appears to facilitate elevations in arterial pressure and cerebral blood flow.

### 4.1 Limitations

The present study has several limitations. First, although we measured MCAV_mean_ as an index of cerebral blood flow, several recent studies using magnetic resonance imaging have reported that the middle cerebral artery dilates in response to hypercapnia (Al‐Khazraji et al., 2019; Coverdale et al., 2014). Therefore, the assessment of cerebrovascular responses to hypercapnia based on MCAV_mean_ may underestimate actual changes. Second, the MCAV_mean_ response observed in the present study does not simply reflect a generalized cerebral artery response. However, given that CO_2_ reactivity in the hypercapnic range is similar in the middle, posterior, and basilar cerebral arteries (Sato et al., 2012; Willie et al., 2012), our MCAV_mean_ results may be applicable to all three of these arteries. Third, we set the *f*_R_ at 60 breaths min^−1^ during CO_2_ inhalation in the normocapnia trial so that the participants could easily match the timing of their kicking and breathing, and V_E_ would be the same in both trials. As a result, the *f*_R_ during CO_2_ inhalation was significantly higher in the normocapnia trial than in the hypercapnia trial (*p* < 0.05). However, given that the increase in *f*_R_ and the concomitant decrease in V_T_ do not affect muscle sympathetic nerve activity in resting humans (Limberg et al., 2013), the influence of the difference in *f*_R_ on MCAV_mean_ and MAP appears to be negligible. Fourth, we only included three women in this study. Since there is potential for sex‐related differences in cerebral blood flow responses (Barnes, 2017) and/or autonomic regulation of cardiovascular responses (Joyner et al., 2015), our results may not reflect responses in young women. Fifth, we did not assess cutaneous circulation. Thus, we do not know the extent to which cutaneous circulation was involved in our results. Sixth, although CO can contribute to MCAV_mean_ (Ainslie & Duffin, 2009; Ogoh et al., 2005), we do not know how much the increased CO associated with hypercapnia increased MCAV_mean_ in the hypercapnia trial. Finally, while the hypercapnia‐induced decrease in LVC observed in the present study was statistically significant, the magnitude was relatively small. This small reduction in the LVC may not be physiologically meaningful.

### 4.2 Conclusion

In summary, MCAV_mean_ and CVCi during CO_2_ inhalation were higher in the hypercapnia trial than in the normocapnia trial. By contrast, LVC was decreased by hypercapnia. These results suggest that, during dynamic exercise, hypercapnia‐induced vasomotion differs between the cerebral circulation and active skeletal muscles. These differential vascular responses may, in part, contribute to a preferential rise in cerebral blood flow.

## AUTHOR CONTRIBUTIONS

S.M., M.I., K.D., N.F., and T.N. conceived and designed experiments. S.M., K.D., R.M., and M.S. performed experiments. S.M., K.D., and R.M. analyzed data. All authors interpreted results of experiments. S.M. prepared figures and drafted the manuscript. All authors edited and revised the manuscript. All authors approved the final version of the manuscript.

## ETHICS STATEMENT

This study was approved by the Human Subjects Committee of the University of Tsukuba (no. 020–74) and was carried out in accordance with the Declaration of Helsinki. Written informed consent was obtained from all participants prior to their participation in the study.

## References

[phy215274-bib-0001] Aaslid, R. , Lindegaard, K. F. , Sorteberg, W. , & Nornes, H. (1989). Cerebral autoregulation dynamics in humans. Stroke, 20, 45–52. 10.1161/01.STR.20.1.45 2492126

[phy215274-bib-0002] Ainslie, P. N. , Ashmead, J. C. , Ide, K. , Morgan, B. J. , & Poulin, M. J. (2005). Differential responses to CO_2_ and sympathetic stimulation in the cerebral and femoral circulations in humans. Journal of Physiology, 566, 613–624. 10.1113/jphysiol.2005.087320 PMC1464750 15890697

[phy215274-bib-0003] Ainslie, P. N. , & Duffin, J. (2009). Integration of cerebrovascular CO_2_ reactivity and chemoreflex control of breathing: mechanisms of regulation, measurement, and interpretation. American Journal of Physiology: Regulatory, Integrative and Comparative Physiology, 296, R1473–R1495. 10.1152/ajpregu.91008.2008 19211719

[phy215274-bib-0004] Al‐Khazraji, B. K. , Shoemaker, L. N. , Gati, J. S. , Szekeres, T. , & Shoemaker, J. K. (2019). Reactivity of larger intracranial arteries using 7 T MRI in young adults. Journal of Cerebral Blood Flow and Metabolism, 39, 1204–1214. 10.1177/0271678X18762880 29513623 PMC6668520

[phy215274-bib-0005] Barnes, J. N. (2017). Sex‐specific factors regulating pressure and flow. Experimental Physiology, 102, 1385–1392. 10.1113/EP086531 28799254 PMC5665704

[phy215274-bib-0006] Battisti‐Charbonney, A. , Fisher, J. , & Duffin, J. (2011). The cerebrovascular response to carbon dioxide in humans. Journal of Physiology, 589, 3039–3048. 10.1113/jphysiol.2011.206052 PMC3139085 21521758

[phy215274-bib-0007] Borg, G. A. (1982). Psychophysical bases of perceived exertion. Medicine and Science in Sports and Exercise, 14, 377–381. 10.1249/00005768-198205000-00012 7154893

[phy215274-bib-0008] Brys, M. , Brown, C. M. , Marthol, H. , Franta, R. , & Hilz, M. J. (2003). Dynamic cerebral autoregulation remains stable during physical challenge in healthy persons. American Journal of Physiology. Heart and Circulatory Physiology, 285, H1048–H1054. 10.1152/ajpheart.00062.2003 12915389

[phy215274-bib-0009] Chin, L. M. , Heigenhauser, G. J. , Paterson, D. H. , & Kowalchuk, J. M. (2013). Effect of voluntary hyperventilation with supplemental CO_2_ on pulmonary O_2_ uptake and leg blood flow kinetics during moderate‐ intensity exercise. Experimental Physiology, 98, 1668–1682. 23975901 10.1113/expphysiol.2013.074021

[phy215274-bib-0010] Coverdale, N. S. , Gati, J. S. , Opalevych, O. , Perrotta, A. , & Shoemaker, J. K. (2014). Cerebral blood flow velocity underestimates cerebral blood flow during modest hypercapnia and hypocapnia. Journal of Applied Physiology, 117, 1090–1096. 10.1152/japplphysiol.00285.2014 25012027

[phy215274-bib-0011] Dobashi, K. , Fujii, N. , Watanabe, K. , Tsuji, B. , Sasaki, Y. , Fujimoto, T. , Tanigawa, S. , & Nishiyasu, T. (2017). Effect of voluntary hyperventilation or moderate hypoxia on metabolic and heart rate responses during high‐intensity intermittent exercise. European Journal of Applied Physiology, 117, 1573–1583. 28527012 10.1007/s00421-017-3646-5

[phy215274-bib-0012] Faraci, F. M. , & Heistad, D. D. (1998). Regulation of cerebral circulation: Role of endothelium and potassium channels. Physiological Reviews, 78, 53–97. 9457169 10.1152/physrev.1998.78.1.53

[phy215274-bib-0013] Fujii, N. , Fujimoto, T. , Cao, Y. , Dobashi, K. , Matsutake, R. , Amano, T. , Watanabe, K. , & Nishiyasu, T. (2021). Caffeine Exacerbates Hyperventilation and Reductions in Cerebral Blood Flow in Physically Fit Males Exercising in the Heat. Medicine and Science in Sports and Exercise, 53, 845–852. 33044440 10.1249/MSS.0000000000002537

[phy215274-bib-0014] Fujii, N. , Kashihara, M. , Kenny, G. P. , Honda, Y. , Fujimoto, T. , Cao, Y. , & Nishiyasu, T. (2019). Carotid chemoreceptors have a limited role in mediating the hyperthermia‐induced hyperventilation in exercising humans. Journal of Applied Physiology, 126, 305–313. 10.1152/japplphysiol.00562.2018 30382804

[phy215274-bib-0015] Gellhorn, E. , & Steck, I. E. (1938). The effect of the inhalation of gases with a low oxygen and an increased carbon dioxide tension on the peripheral blood flow in man. American Journal of Physiology, 124, 735–741. 10.1152/ajplegacy.1938.124.3.735

[phy215274-bib-0016] Guyenet, P. G. , Stornetta, R. L. , Abbott, S. B. , Depuy, S. D. , Fortuna, M. G. , & Kanbar, R. (2010). Central CO_2_ chemoreception and integrated neural mechanisms of cardiovascular and respiratory control. Journal of Applied Physiology, 108, 995–1002. 20075262 10.1152/japplphysiol.00712.2009 PMC2853202

[phy215274-bib-0017] Ichinose, M. , Matsumoto, M. , Fujii, N. , Yoshitake, N. , & Nishiyasu, T. (2018). Voluntary apnea during dynamic exercise activates the muscle metaboreflex in humans. American Journal of Physiology. Heart and Circulatory Physiology, 314, H434–H442. 10.1152/ajpheart.00367.2017 29101169

[phy215274-bib-0018] Ichinose, M. , & Nishiyasu, T. (2005). Muscle metaboreflex modulates the arterial baroreflex dynamic effects on peripheral vascular conductance in humans. American Journal of Physiology. Heart and Circulatory Physiology, 288, H1532–H1538. 10.1152/ajpheart.00673.2004 15576444

[phy215274-bib-0019] Ide, K. , Eliasziw, M. , & Poulin, M. J. (2003). Relationship between middle cerebral artery blood velocity and end‐tidal PCO_2_ in the hypocapnic‐hypercapnic range in humans. Journal of Applied Physiology, 95, 129–137. 10.1152/japplphysiol.01186.2002 19278048

[phy215274-bib-0020] Jouett, N. P. , Watenpaugh, D. E. , Dunlap, M. E. , & Smith, M. L. (2015). Interactive effects of hypoxia, hypercapnia and lung volume on sympathetic nerve activity in humans. Experimental Physiology, 100, 1018–1029. 10.1113/EP085092 26132990

[phy215274-bib-0021] Joyner, M. J. , Barnes, J. N. , Hart, E. C. , Wallin, B. G. , & Charkoudian, N. (2015). Neural control of the circulation: how sex and age differences interact in humans. Comprehensive Physiology, 5, 193–215. 25589269 10.1002/cphy.c140005 PMC4459710

[phy215274-bib-0022] Kontos, H. A. , Richardson, D. W. , & Patterson, J. L. Jr (1968b). Vasodilator effect of hypercapnic acidosis on human forearm blood vessels. American Journal of Physiology, 215, 1403–1405. 10.1152/ajplegacy.1968.215.6.1403 5723001

[phy215274-bib-0023] Kontos, H. A. , Richardson, W. D. , & Patterson, J. L. Jr (1968a). Roles of hypercapnia and acidosis in the vasodilator response to hypercapnic acidosis. American Journal of Physiology, 215, 1406–1408. 10.1152/ajplegacy.1968.215.6.1406 5723002

[phy215274-bib-0024] Laughlin, M. H. , Korthuis, R. J. , Duncker, D. J. , & Bache, R. J. (1996). Control of blood flow to cardiac and skeletal muscle during exercise. Handbook of physiology, section 12, exercise: regulation and integration of multiple systems, chapter 16, (pp. 705–769). Am Physiol Soc.

[phy215274-bib-0025] LeMarbre, G. , Stauber, S. , Khayat, R. N. , Puleo, D. S. , Skatrud, J. B. , & Morgan, B. J. (2003). Baroreflex‐induced sympathetic activation does not alter cerebrovascular CO_2_ responsiveness in humans. Journal of Physiology, 551, 609–616. 10.1113/jphysiol.2003.046987 PMC2343219 12844511

[phy215274-bib-0026] Lennox, W. G. , & Gibbs, E. L. (1932). The blood flow in the brain and the leg of man, and the changes induced by alteration of blood gases. Journal of Clinical Investigation, 11, 1155–1177. 10.1172/JCI100470 PMC435872 16694095

[phy215274-bib-0027] Limberg, J. K. , Morgan, B. J. , Schrage, W. G. , & Dempsey, J. A. (2013). Respiratory influences on muscle sympathetic nerve activity and vascular conductance in the steady state. American Journal of Physiology. Heart and Circulatory Physiology, 304, H1615–H1623. 10.1152/ajpheart.00112.2013 23585141 PMC3680774

[phy215274-bib-0028] Maggio, P. , Salinet, A. S. , Panerai, R. B. , & Robinson, T. G. (2013). Does hypercapnia‐induced impairment of cerebral autoregulation affect neurovascular coupling? A functional TCD study. Journal of Applied Physiology, 115, 491–497. 10.1152/japplphysiol.00327.2013 23743398 PMC3742941

[phy215274-bib-0029] Moreira, T. S. , Takakura, A. C. , Colombari, E. , & Guyenet, P. G. (2006). Central chemoreceptors and sympathetic vasomotor outflow. Journal of Physiology, 577, 369–386. 10.1113/jphysiol.2006.115600 PMC2000682 16901945

[phy215274-bib-0030] Narkiewicz, K. , Pesek, C. A. , van de Borne, P. , Kato, M. , & Somers, V. K. (1999). Enhanced sympathetic and ventilatory responses to central chemoreflex activation in heart failure. Circulation, 100, 262–267. 10.1161/01.CIR.100.3.262 10411850

[phy215274-bib-0031] Nishiyasu, T. , Tsukamoto, R. , Kawai, K. , Hayashi, K. , Koga, S. , & Ichinose, M. (2012). Relationships between the extent of apnea‐induced bradycardia and the vascular response in the arm and leg during dynamic two‐legged knee extension exercise. American Journal of Physiology‐Heart and Circulatory Physiology, 302, H864–H871. 10.1152/ajpheart.00413.2011 22159992

[phy215274-bib-0032] Ogoh, S. , Ainslie, P. N. , & Miyamoto, T. (2009). Onset responses of ventilation and cerebral blood flow to hypercapnia in humans: Rest and exercise. Journal of Physiology, 106, 880–886. 10.1152/japplphysiol.91292.2008 PMC2660255 19131474

[phy215274-bib-0033] Ogoh, S. , Brothers, R. M. , Barnes, Q. , Eubank, W. L. , Hawkins, M. N. , Purkayastha, S. , O‐Yurvati, A. , & Raven, P. B. (2005). The effect of changes in cardiac output on middle cerebral artery mean blood velocity at rest and during exercise. Journal of Physiology, 569, 697–704. 10.1113/jphysiol.2005.095836 PMC1464249 16210355

[phy215274-bib-0034] Ogoh, S. , Hayashi, N. , Inagaki, M. , Ainslie, P. N. , & Miyamoto, T. (2008). Interaction between the ventilatory and cerebrovascular responses to hypo‐ and hypercapnia at rest and during exercise. Journal of Physiology, 586, 4327–4338. 10.1113/jphysiol.2008.157073 PMC2652171 18635644

[phy215274-bib-0035] Panerai, R. B. , Deverson, S. T. , Mahony, P. , Hayes, P. , & Evans, D. H. (1999). Effect of CO_2_ on dynamic cerebral autoregulation measurement. Physiological Measurement, 20, 265–275. 10475580 10.1088/0967-3334/20/3/304

[phy215274-bib-0036] Rasmussen, P. , Stie, H. , Nielsen, B. , & Nybo, L. (2006). Enhanced cerebral CO_2_ reactivity during strenuous exercise in man. European Journal of Applied Physiology, 96, 299–304. 10.1007/s00421-005-0079-3 16284788

[phy215274-bib-0037] Rowell, L. B. (1993). Control of regional blood flow during dynamic exercise. In: Human cardiovascular control, section 1, chapter 6, (pp. 204–206). Oxford University Press.

[phy215274-bib-0038] Sato, K. , Sadamoto, T. , Hirasawa, A. , Oue, A. , Subudhi, A. W. , Miyazawa, T. , & Ogoh, S. (2012). Differential blood flow responses to CO_2_ in human internal and external carotid and vertebral arteries. Journal of Physiology, 590, 3277–3290. 10.1113/jphysiol.2012.230425 PMC3459042 22526884

[phy215274-bib-0039] Schultz, H. D. , & Sun, S. Y. (2000). Chemoreflex function in heart failure. Heart Failure Reviews, 5, 45–56. 16228915 10.1023/A:1009846123893

[phy215274-bib-0040] Simmons, G. H. , Padilla, J. , Young, C. N. , Wong, B. J. , Lang, J. A. , Davis, M. J. , Laughlin, M. H. , & Fadel, P. J. (2011). Increased brachial artery retrograde shear rate at exercise onset is abolished during prolonged cycling: Role of thermoregulatory vasodilation. Journal of Applied Physiology, 110, 389–397. 21088203 10.1152/japplphysiol.00936.2010 PMC3043792

[phy215274-bib-0041] Somers, V. K. , Mark, A. L. , & Abboud, F. M. (1991). Interaction of baroreceptor and chemoreceptor reflex control of sympathetic nerve activity in normal humans. Journal of Clinical Investigation, 87, 1953–1957. 10.1172/JCI115221 PMC296947 2040688

[phy215274-bib-0042] Somers, V. K. , Mark, A. L. , Zavala, D. C. , & Abboud, F. M. (1989). Contrasting effects of hypoxia and hypercapnia on ventilation and sympathetic activity in humans. Journal of Applied Physiology, 67, 2101–2106. 10.1152/jappl.1989.67.5.2101 2513316

[phy215274-bib-0043] Subudhi, A. W. , Olin, J. T. , Dimmen, A. C. , Polaner, D. M. , Kayser, B. , & Roach, R. C. (2011). Does cerebral oxygen delivery limit incremental exercise performance? Journal of Applied Physiology, 111, 1727–1734. 10.1152/japplphysiol.00569.2011 21921244 PMC3233884

[phy215274-bib-0044] Toledo, C. , Andrade, D. , Lucero, C. , Schultz, H. , Marcus, N. , Retamal, M. , Madrid, C. , & Rio, R. (2017). Contribution of peripheral and central chemoreceptors to sympatho‐excitation in heart failure. Journal of Physiology, 595, 43–51. 10.1113/JP272075 PMC5199744 27218485

[phy215274-bib-0045] Vantanajal, J. S. , Ashmead, J. C. , Anderson, T. J. , Hepple, R. T. , & Poulin, M. J. (2007). Differential sensitivities of cerebral and brachial blood flow to hypercapnia in humans. Journal of Applied Physiology, 102, 87–93. 10.1152/japplphysiol.00772.2006 17023571

[phy215274-bib-0046] Wallace, M. , Hashim, Y. Z. , Wingfield, M. , Culliton, M. , McAuliffe, F. , Gibney, M. J. , & Brennan, L. (2010). Effects of menstrual cycle phase on metabolomic profiles in premenopausal women. Human Reproduction, 25, 949–956. 10.1093/humrep/deq011 20150174

[phy215274-bib-0047] Wan, H.‐Y. , Weavil, J. C. , Thurston, T. S. , Georgescu, V. P. , Hureau, T. J. , Bledsoe, A. D. , Buys, M. J. , Jessop, J. E. , Richardson, R. S. , & Amann, M. (2020). The exercise pressor reflex and chemoreflex interaction: cardiovascular implications for the exercising human. Journal of Physiology, 598(12), 2311–2321. 10.1113/JP279456 PMC7720421 32170732

[phy215274-bib-0048] Wesseling, K. H. , Jansen, J. R. , Settels, J. J. , & Schreuder, J. J. (1993). Computation of aortic flow from pressure in humans using a nonlinear, three‐element model. Journal of Applied Physiology, 74, 2566–2573. 10.1152/jappl.1993.74.5.2566 8335593

[phy215274-bib-0049] Willie, C. K. , Macleod, D. B. , Shaw, A. D. , Smith, K. J. , Tzeng, Y. C. , Eves, N. D. , Ikeda, K. , Graham, J. , Lewis, N. C. , Day, T. A. , & Ainslie, P. N. (2012). Regional brain blood flow in man during acute changes in arterial blood gases. Journal of Physiology, 590, 3261–3275. 10.1113/jphysiol.2012.228551 PMC3459041 22495584

[phy215274-bib-0050] Xie, A. , Skatrud, J. B. , Puleo, D. S. , & Morgan, B. J. (2001). Exposure to hypoxia produces long‐lasting sympathetic activation in humans. Journal of Applied Physiology, 91, 1555–1562. 10.1152/jappl.2001.91.4.1555 11568136

